# Checkpoint Activation of an Unconventional DNA Replication Program in *Tetrahymena*

**DOI:** 10.1371/journal.pgen.1005405

**Published:** 2015-07-28

**Authors:** Pamela Y. Sandoval, Po-Hsuen Lee, Xiangzhou Meng, Geoffrey M. Kapler

**Affiliations:** 1 Interdisciplinary Program in Genetics, Texas A&M University, College Station, Texas, United States of America; 2 Department of Molecular and Cellular Medicine, Texas A&M University Health Science Center, College Station, Texas, United States of America; 3 Department of Biochemistry and Biophysics, Texas A&M University, College Station, Texas, United States of America; University of Vienna, AUSTRIA

## Abstract

The intra-S phase checkpoint kinase of metazoa and yeast, ATR/MEC1, protects chromosomes from DNA damage and replication stress by phosphorylating subunits of the replicative helicase, MCM2-7. Here we describe an unprecedented ATR-dependent pathway in *Tetrahymena thermophila* in which the essential pre-replicative complex proteins, Orc1p, Orc2p and Mcm6p are degraded in hydroxyurea-treated S phase cells. Chromosomes undergo global changes during HU-arrest, including phosphorylation of histone H2A.X, deacetylation of histone H3, and an apparent diminution in DNA content that can be blocked by the deacetylase inhibitor sodium butyrate. Most remarkably, the cell cycle rapidly resumes upon hydroxyurea removal, and the entire genome is replicated prior to replenishment of ORC and MCMs. While stalled replication forks are elongated under these conditions, DNA fiber imaging revealed that most replicating molecules are produced by new initiation events. Furthermore, the sole origin in the ribosomal DNA minichromosome is inactive and replication appears to initiate near the rRNA promoter. The collective data raise the possibility that replication initiation occurs by an ORC-independent mechanism during the recovery from HU-induced replication stress.

## Author Summary

DNA damage and replication stress activate cell cycle checkpoint responses that protect the integrity of eukaryotic chromosomes. A well-conserved response involves the reversible phosphorylation of the replicative helicase, MCM2-7, which together with the origin recognition complex (ORC) dictates when and where replication initiates in chromosomes. The central role of ORC and MCMs in DNA replication is illustrated by the fact that small changes in abundance of these pre-replicative complex (pre-RC) components are poorly tolerated from yeast to humans. Here we describe an unprecedented replication stress checkpoint response in the early branching eukaryote, *Tetrahymena thermophila*, that is triggered by the depletion of dNTP pools with hydroxyurea (HU). Instead of transiently phosphorylating MCM subunits, ORC and MCM proteins are physically degraded in HU-treated *Tetrahymena*. Unexpectedly, upon HU removal the genome is completely and effortlessly replicated prior to replenishment of ORC and MCM components. Using DNA fiber imaging and 2D gel electrophoresis, we show that ORC-dependent mechanisms are bypassed during the recovery phase to produce bidirectional replication forks throughout the genome. Our findings suggest that *Tetrahymena* enlists an alternative mechanism for replication initiation, and that the underlying process can operate on a genome-wide scale.

## Introduction

A major challenge of the cell cycle is to faithfully transmit chromosomes to daughter cells. This is accomplished through the replication and segregation of chromosomes during the respective S and M phases. The integrity of chromosomes is under constant assault from extrinsic and intrinsic sources that directly damage DNA or generate roadblocks for the replication machinery. The resulting DNA damage and replication stress can irreparably harm chromosomes. Checkpoint pathways have evolved to combat these problems, arresting the cell cycle when thresholds are exceeded. The phosphatidylinositol-3-OH kinase family members ATM (Ataxia Telangiectasia Mutated) and ATR (ATM-and Rad3-related) function as apical kinases in signal transduction pathways that inhibit DNA replication and promote DNA repair [1]. ATM is activated by double strand breaks (DSBs) and arrests the cell cycle at the G1/S border, while ATR monitors replication stress and other types of DNA damage during S phase. ATR is recruited to exposed stretches of single strand DNA through association with its binding partner, ATRIP. A key target of ATR is the MCM2-7 complex. Phosphorylation of the replicative helicase suppresses fork elongation, and prevents new origins from firing [2–4]. Once the source of stress is removed, fork elongation rates are restored and new initiation events occur at late replicating or dormant origins [5–7].

While the proteins that elicit checkpoint responses are conserved, there are fundamental differences in how eukaryotes deal with DNA damage. For example, while hydroxyurea (HU) inhibits initiation from late firing origins and slows replication fork elongation in *S*. *cerevisiae* [8], mammalian ATR arrests both processes [9,10]. Most late firing *S*. *pombe* origins are regulated by a checkpoint-independent mechanism [11]. Moreover, the contribution of error prone polymerases and homologous recombination to lesion bypass/repair varies considerably across the eukaryotic lineage. *Arabidopsis thaliana* elicits a unique response to double strand breaks, in which cell cycle arrest is averted. Instead, the ATR regulated, plant-specific transcription factor, SOG1, induces genome-wide endoreplication [12].

In this study we explore the intra-S phase checkpoint response in *Tetrahymena thermophila*. As a representative of the Ciliophora lineage, *Tetrahymena* contains two nuclei within a single cytoplasm: the diploid germ line micronucleus and the polyploid (45 C) somatic macronucleus [13]. The transcriptionally-silent micronucleus encodes genetic information that is transmitted from parent to progeny during the sexual phase of the life cycle. Its five chromosomes undergo conventional mitosis and meiosis. The actively transcribed macronucleus is polyploid, confers the cellular phenotype and the ~17,000 chromosomes are randomly segregate at each cell division. Since the parental macronucleus is destroyed in progeny, a new macronucleus must be generated. This process involves the extensive reorganization of germ line precursor chromosomes. The five micronuclear chromosomes are processed into ~180 unique macronuclear products that endoreplicate to a copy number of 45 C. The 21 kb ribosomal DNA (rDNA) minichromosome is further amplified to ~9000 C.

Macronuclear chromosomes lack centromeres. While the intrinsic imprecision of amitosis can generate genic imbalances, both DNA content and chromosome copy number are maintained within a narrow range. ‘Excess’ DNA is eliminated in the form of chromatin extrusion bodies [14] and macronuclear chromosomes re-replicate when total DNA content falls below an acceptable minimum [15]. Although the fundamental components for replication initiation (ORC, MCMs, Cdt1) and the DNA damage/replication stress checkpoint response (ATR, Rad51, gamma H2A.X) are conserved in *Tetrahymena*, their roles in genome maintenance are only partially understood.

We previously identified a caffeine-sensitive DNA damage/replication stress checkpoint pathway that is activated by hydroxyurea (HU) and methylmethanesulphonate (MMS) [16]. Inhibition of this pathway results in aberrant macronuclear division and micronuclear genome instability, consistent with a protective role for chromosomes. A single ATR gene was identified (TTHERM_01008650), however, no obvious ATM candidates were detected (Tetrahymena Genome Database (TGD), www.ciliate.org). ATR is required for the reorganization of chromosomes during meiosis as well [17].

Our recent analysis of an ORC1 knockdown strain provided unexpected insights into the regulation of MCM2-7, a major target of ATR [18]. Like the *S*. *cerevisiae* orc2-1 mutant [19], a moderate reduction in *Tetrahymena* Orc1p induces genome instability, but fails to trigger an intra-S phase checkpoint response [18]. In contrast to the budding yeast mutant, replication initiation is unaffected in *Tetrahymena* ORC1 knockdown strains. Instead, the rate of replication fork elongation decreases and Mcm6p levels are reduced, suggesting that the replicative helicase becomes rate limiting. Since the MCM complex is much more abundant than ORC in other eukaryotes [20], this fork elongation defect was unanticipated. Moreover, while moderate reductions in pre-replicative complex (pre-RC) components are poorly tolerated during the *Tetrahymena* vegetative cell cycle, ORC and MCM levels oscillate to a much greater degree during development. Paradoxically, these pre-RC proteins are more abundant early in development when the DNA replication load is comparatively modest, and decline dramatically when the replication load increases [18]. At low ORC concentrations, the rDNA origin is frequently bypassed, raising the possibility that alternative initiation sites (cryptic origins) and/or unconventional mechanisms are called into play.

In the work presented here, we document an unprecedented checkpoint response to HU treatment that begins with the targeted degradation of *Tetrahymena* ORC and MCM subunits. We provide evidence for alterations in macronuclear chromosomes that include global deacetylation of histone H3, and manifest as an apparent time-dependent decrease in DNA content in S phase arrested cells. More remarkably, upon HU removal, DNA synthesis resumes and the entire genome is duplicated prior to replenishment of ORC and MCMs. The rDNA minichromosome is replicated, although its ORC-dependent origin is inactive. Non-rDNA chromosomes also initiate bidirectional DNA replication prior to ORC replenishment. These findings argue for the existence of an alternative mechanism for DNA replication that operates on a genome-wide scale when ORC and MCM proteins are rate limiting.

## Results

### HU and MMS trigger unconventional checkpoint responses

**Fig 1:**
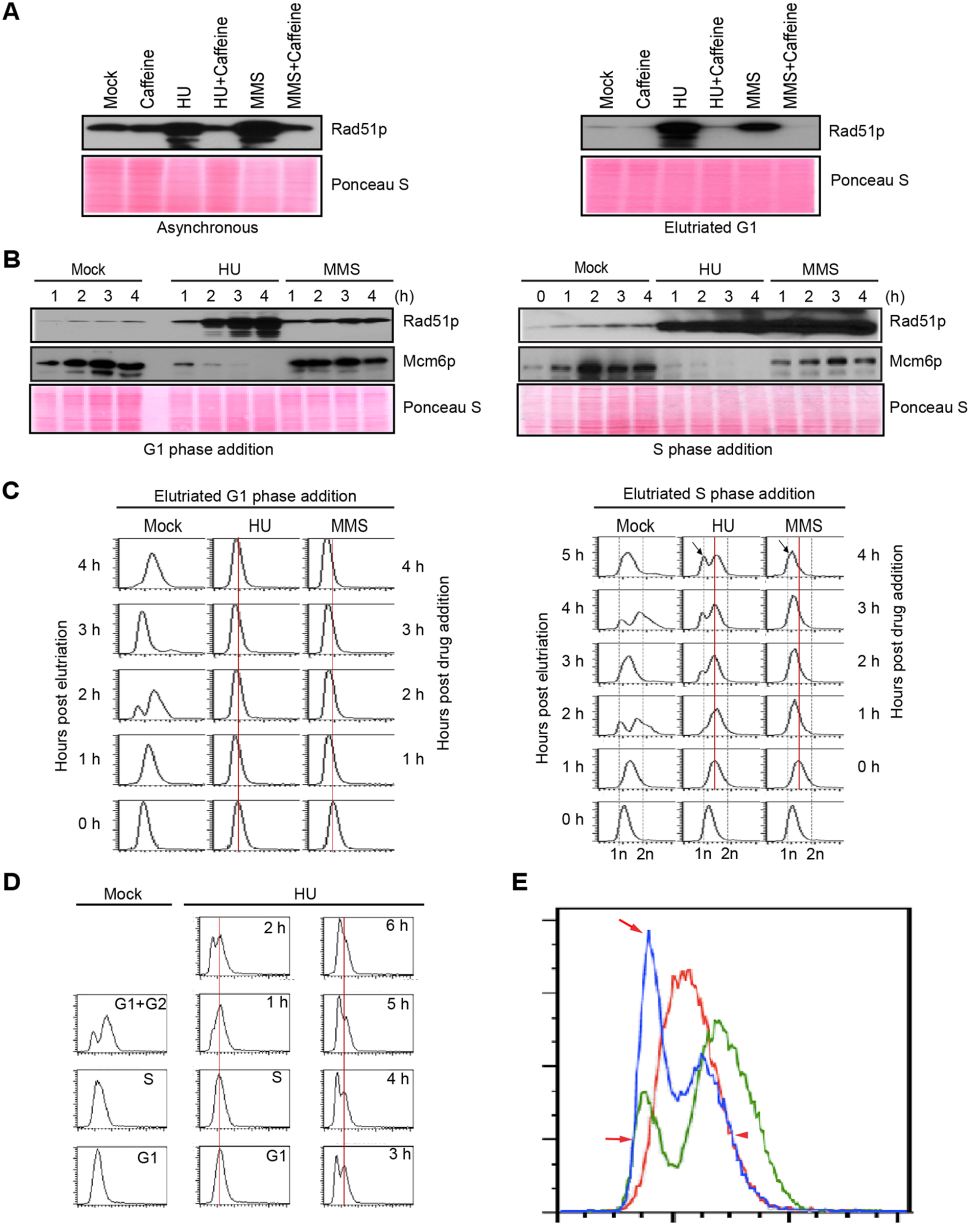
Characterization of HU and MMS induced checkpoint responses. (A) Western blot analysis of Rad51p for asynchronous log phase cultures (left panel) or elutriated G1 phase cells (right panel) treated for 4 h with 20 mM HU, 0.06% MMS, 1 mM caffeine, or in combination. Equivalent amounts of protein were loaded in each lane. Ponceau S staining was used to verify protein transfer and serve as a crude validation for equal protein loading prior to antibody probing. (B) G1/S and intra-S phase checkpoint analysis. Cells were synchronized at the G1/S border by centrifugal elutriation. 20 mM HU or 0.06% MMS were added immediately (G1 phase addition, left panel) or 1 h later (mid-S phase addition, right panel). Western blot analysis was performed with antibodies specific directed against Rad51p and Mcm6p. The zero hour time point (right panel) corresponds to freshly elutriated G1 cells for both the G1 (left panel) and mid-S phase (right panel) treatment regimens. (C) Flow cytometry analysis of cell populations analyzed in B. The vertical red line demarcates the peak at the time of drug addition. The arrow (4 h post drug addition) in cultures treated with HU or MMS during S phase demarcates the newly formed G1-like peak. (D) Flow cytometry profiling during an extended HU treatment of S-phase cells. Left panel: mock-treated cells. Right panel: HU-treated cultures in which elutriated G1 cells were grown for 1 h (to early/mid S phase) prior to addition of 20 mM HU for 0–6 h. (E) Data from HU-treated mid-S phase cells. Control flow cytometry profiles for mock-treated cells in S phase (red line, 1 h after G1 isolation) and G1+ G2 phase (green line, 2 h after G1 isolation). Treated cells cultured with HU for 4 h (blue line, HU treatment beginning 1 h after elutriation). Red arrows: newly formed peak at the G1 position in HU-treated cells (blue profile). Red arrowhead: right shoulder for the cell population at time of HU addition (red profile) and 4 h late (blue profile).

As previously reported, HU and MMS, arrest cell cycle progression and activate a DNA damage/replication stress checkpoint response in *Tetrahymena*, including induction of the double strand break repair protein, Rad51p (Fig 1A, left panel) [16]. Rad51 accumulation is inhibited by the addition of caffeine, consistent with the involvement of an apical PI3-like kinase. Since MMS activates both ATM and ATR in mammals, we used centrifugal elutriation to distinguish between G1/S (ATM-like) and intra-S phase (ATR) checkpoint responses. HU or MMS were added immediately to a homogeneous G1 phase population or 1 h later (midway through S phase). Rad51p levels were elevated ~10-fold and 2-fold, respectively; for HU and MMS treated G1 phase cells relative to mock controls (Fig 1A, right panel, 4 h treatment). To better monitor Rad51p induction, cells were examined at 1 h intervals. Whereas the HU-dependent signal increased continuously over 4 h, the Rad51p level quickly plateaued in MMS-treated cells (Fig 1B, left panel). To address whether this difference is due to a block in progression from G1 to S phase in MMS-treated cells, elutriated cultures were grown to mid-S phase prior to drug addition (Fig 1B, right panel). HU and MMS elicited strong and equivalent Rad51p responses in S phase treated cells. These data argue for the existence of distinct ATM-like (G1) and ATR (S phase) checkpoint responses.

Flow cytometry was used to examine the effect of HU and MMS on bulk DNA synthesis in G1 and S phase treated cells. As expected, DNA replication was rapidly inhibited by either drugs, regardless of the time of drug addition. Whereas the DNA profile of G1 arrested cells remained constant over the examined 4 h interval (Fig 1C, left panel), the profile of S phase treated cells changed in an unanticipated way. An additional peak migrating at the G1 position appeared 1 h after HU addition and became more prominent over time (Fig 1C, right panel, arrow). Two distinct sub-populations were generated, one arrested in S phase and the other displaying an apparent G1 DNA content. MMS treatment did not generate this bimodal distribution (Fig 1C, right). Instead the S phase peak gradually shifted to the left, suggesting that DNA content within the entire population had decreased. Both results were highly reproducible (n = 8). These flow cytometry profiles were recapitulated in cells synchronized by starvation and re-feeding, with the following variation: bimodal peaks were occasionally observed in MMS-treated S phase cells (S1B Fig, arrow).

To investigate this phenomenon further, HU studies were expanded on elutriated S phase cultures. Mock-treated G1, S and G2 phase populations (Fig 1D, left panel) were used as reference points to determine whether HU-treated S phase cells proceeded through G2 prior to generating the G1 profile. The data collected over 6 h are not consistent with this model (Fig 1D). The DNA profile of S phase arrested cells did not shift to the right (more DNA) prior to formation of the G1 peak. In another experiment, we overlaid the 4 h HU profile with mock-treated G1, S and G2 DNA reference points (Fig 1E). Two features stand out. First, the right shoulder of the HU-treated population (blue profile, smaller peak) aligned precisely with the right shoulder of the S phase peak (red profile; T = 0 h for HU addition), indicating that that DNA content did not increase in this HU-arrested subpopulation. Second, the left peak and shoulder of the HU-treated profile (blue profile, larger peak, arrows) aligned with the G1 profile (green profile). No sub-G1 peak was observed, consistent with the idea that cells did not exit S phase and divide precociously. A subsequent flow cytometry analysis at 10 min intervals showed no evidence for a G2 peak (S2 Fig). The collective data suggest that the DNA content of HU-treated *Tetrahymena* neither remains constant nor gradually increases, as has been reported for *S*. *pombe* [21] and *S*. *cerevisiae* [8], respectively. Instead, the DNA content in the *Tetrahymena* macronucleus appears to decrease over time.

### HU and MMS do not promote precocious cell division or macronuclear chromatin elimination

**Fig 2:**
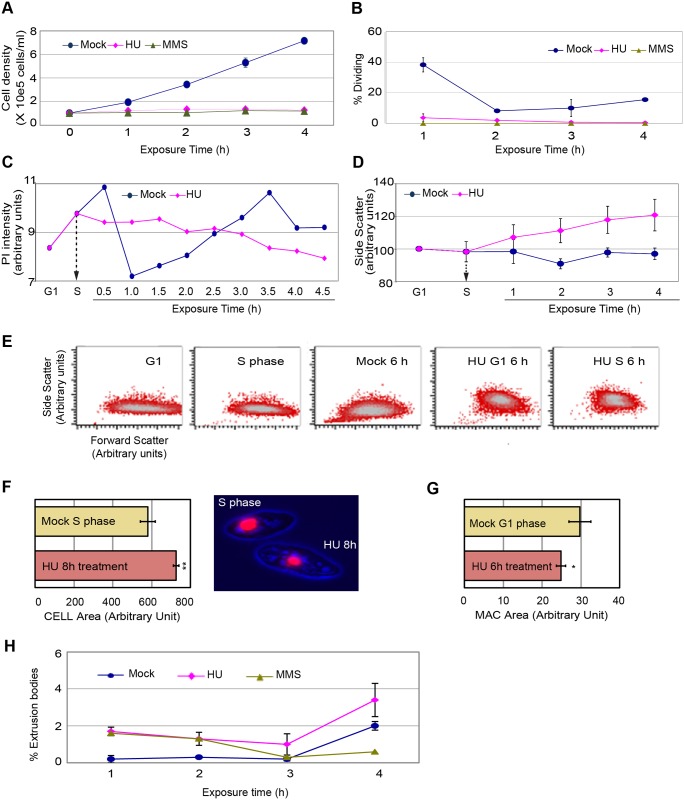
Physical characterization of cells in mock, 20 mM HU and 0.06% MMS treated cultures. A G1 cell population was obtained by centrifugal elutriation and propagated for 1 h (mid-S phase) prior to the addition of HU or MMS. (A) Cell density analysis by direct counting on a hemocytometer). (B) Visualization of the cytokinetic furrow (a marker for cell division) in fixed DAPI-stained cells. Approximately 40% of mock-treated cells generated a cytokinetic furrow 2 h after G1 phase isolation (1 h after HU or MMS addition in treated samples). T = 4 h, mock versus HU: (student T test) two-paired P value 0.0002); T = 4 h, mock versus MMS: (student T test) two-paired P value <0.0001). (C) Plot of DNA content (PI, propidium iodide) in mock and HU-treated cells as a function of exposure time. Elutriated G1 phase cells were treated with 20 mM HU beginning 1 h after isolation (vertical arrow, mid-S phase addition) and samples were collected every 30 min for 4.5 h. 30,000 cells were scored at each time point. (D) Analysis of flow cytometry side scatter (SSC) in mock and HU-treated cells. HU was added during S phase and samples were collected at 1 h intervals and subjected to statistical analysis. The results of three independent experiments (n = 3) are compiled. (E) Visual representation of flow cytometry side scatter (SSC) and forward scatter (FSC) in cells synchronized by centrifugal elutriation. Controls: mock-treated G1 cells (T = 0 h after elutriation), mock-treated S phase cells (T = 1 h after elutriation), and mock-treated cells 6 h after elutriation. Experimental samples: 6 h HU exposure beginning in G1 phase (0 h after elutriation; (HU/G1, 6 h), or in S phase (1 h after elutriation (HU/S, 6 h). The higher complexity seen in HU-treated cells was not observed at any stage of the cell cycle in untreated controls (see S3 Fig). (F) Left panel: the total cell area for 100 microscopic cell images was measured in mock S phase and HU-treated cells. The average area was determined and subjected to statistical analysis. A 30% increase in cell size was observed in HU-treated cells (T = 8 h HU versus mock S phase). Student T test two-paired P value <0.0001 (highly significant). Right panel: representative micrograph illustrating the size difference between mock-treated S phase cells and S phase cells arrested with HU for 8 h. (G) Total macronuclear area for 100 microscopic cell images was measured in mock and HU-treated cells. The average area was determined and subjected to statistical analysis. A 20% decrease in the size of the macronucleus was observed in HU-treated cells (T = 6 h HU versus mock G1 phase cells). Student T test two-paired P value <0.1 (significant). (H) Production of macronuclear extrusion bodies. DAPI staining was used to identify cells with DNA masses that were not associated with the micro- or macronucleus (macronuclear extrusion bodies). T = 4 h, mock versus HU. Student T test, two-paired P value: 0.2249 (not significant).

Two mechanisms for decreasing macronuclear DNA content have been previously reported: asymmetric macronuclear division [22,23] and elimination of ‘excess’ DNA in the form of chromatin extrusion bodies (CEBs) [24]. Multiple criteria were used to examine these and other possibilities in HU or MMS-treated S phase cells. First, we monitored cell number. In contrast to mock-treated controls, cell density did not increase in HU or MMS treated cultures (Fig 2A). Second, we visually assessed cell division. Unlike mock-treated controls, cytokinesis was not observed in HU-treated populations (Fig 2B). Third, we quantified flow cytometry data over time. HU-treated S phase cells displayed a gradual, steady decline in apparent DNA content (Fig 2C, red profile), while the DNA content of mock-treated cells oscillated in conjunction with cell division (Fig 2C, blue profile). Fourth, we assessed cytoplasmic complexity/granularity. Consistent with the absence of cell division, the side scatter parameter increased substantially in the flow cytometry profile of HU-treated cells (Fig 2D, red graph, and S3C Fig) relative to mock-treated controls (Fig 2D, blue graph, and S3A Fig). The increased side scatter in HU-treated cells exceeded values generated at any stage of the cell cycle in mock-treated controls, and was not dependent on the time of drug addition (Figs 2E, S3B and S3C, compare HU addition to G1 or S phase cultures). Fifth, cell size and macronuclear size were quantified in DAPI-stained samples. Consistent with cell division arrest, HU-treated cells were on average 1.3X larger than mock-treated S phase controls (Fig 2F graph, P <0.001; highly significant). Conversely, the size of the macronucleus appeared smaller (Fig 2F, representative micrograph).

To better assess the relationship between HU treatment and macronuclear size, mock-treated G1 phase cells were used as our reference point. While HU-treated mid-S phase cells were larger than G1 phase mock controls, their macronuclei were on average 20% smaller (Fig 2G, P<0.1; significant) (nuclear/cytoplasmic ratio: 0.66X). Since, no sub-G1 DNA peak appears in HU-treated S phase cells (Fig 1E), these data suggest that the global architecture of the macronucleus is somehow altered (see below). Finally, DAPI was used to visually assess chromatin exclusion body (CEB) formation (Fig 2H). The modest increase in CEBs in HU-treated cells was statistically insignificant, and cannot account for the large sub-population of cells with diminished (G1) DNA content. The collective data demonstrate that HU and MMS-treated *Tetrahymena* do not undergo cell division or modulate their DNA content through general or ciliate-specific mechanism.

### Stabilization of nascent strands and down-regulation of histone H3 acetylation

**Fig 3:**
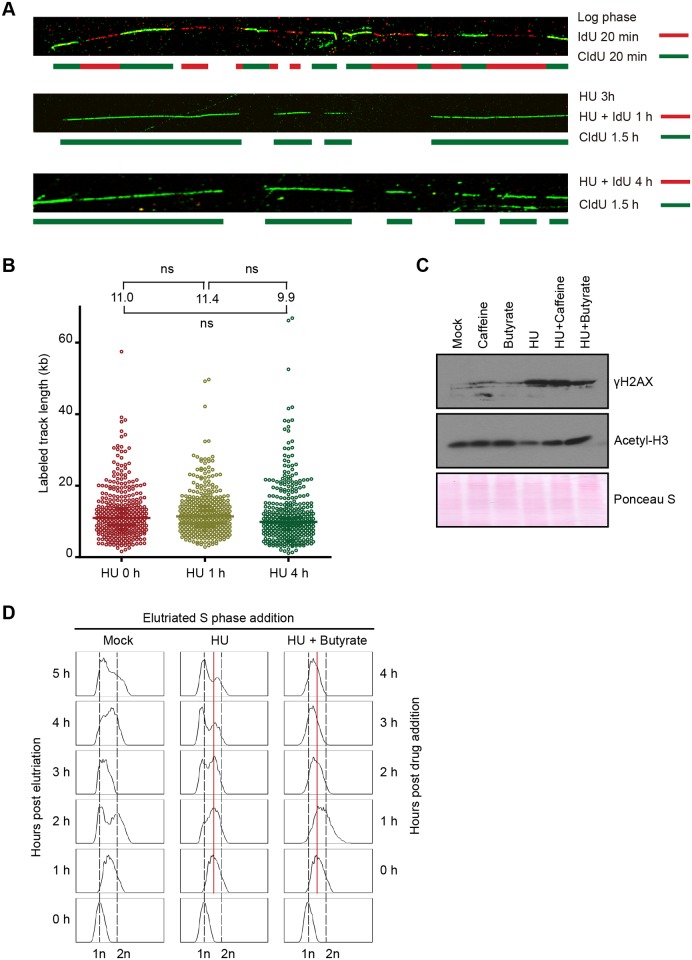
Effect of HU on DNA replication and acetylation of histone H3. (A) DNA fiber analysis was performed in the presence or absence of HU to assess nascent strand synthesis and stability. Cells were synchronized at the G1/S border by starvation and re-feeding, and cultured in the presence of 20 mM HU and 400 μM IdU for 4 h, or 20 mM HU for 3 h, then 20 mM HU plus 400 μM IdU for 1 h. HU and IdU were removed, and cells were further cultured for 90 min in media containing 100 μM CldU. DNA fibers were visualized as described in Materials and Methods (IdU, red; CldU, green). As a control, an untreated log phase culture was sequentially pulse labeled for 20 min in media containing IdU and CldU. (B) Mid-S phase cells were pulse labeled with 100 μM CldU for 10 min prior to the addition of HU, and DNA fibers were prepared 0, 1 and 4 h after HU treatment. The length of individual labeled DNA tracts was plotted and the median tract length was calculated as subjected to statistical analysis. (C) Western blot analysis with phospho-gamma H2A.X and acetylated histone H3 antibodies. Synchronized starved/re-fed cells were treated for 4 h with the indicated drug or drug combination (20 mM HU, 1mM caffeine, 50 mM sodium butyrate) and cells were lysed in SDS loading buffer. Twenty micrograms of protein was loaded in each lane. Ponceau S staining was used to verify protein transfer and served as a crude validation for equal protein loading prior to antibody probing. (D) Flow cytometry profiles of elutriated cells treated with 20 mM HU or 20 mM HU and 50 mM sodium butyrate for 1–4 h.

Two additional mechanisms were considered to explain the apparent decrease in DNA content in S phase arrested cells: active degradation of DNA and global compaction of chromatin. DNA fiber analysis was used to examine DNA synthesis and determine the fate of pulse labeled DNA over time. As a starting point, we re-fed starved, G1-arrested cells with media containing HU for 4 h. HU-treated cells were simultaneously labeled with IdU for 1–4 h, and DNA fibers were examined for incorporation of IdU into nascent strands. Whereas cells in an asynchronous population generated long IdU tracts in a 20 min labeling (Fig 3A, upper image, red tracts), no incorporation was detected in HU-treated cells, regardless of the timing or duration of labeling (Fig 3A, middle (1 h IdU) and lower (4 h IdU) images). However, CldU was readily incorporated following HU removal (green tracts). We conclude that HU arrests replication forks rather than slowing the rate of fork elongation.

To examine the fate of nascent strands in HU-treated cells, synchronized mid-S phase cells were pulse labeled with IdU for 10 min prior to HU addition, and DNA fibers were prepared 1 and 4 h later. As a control, cells were collected directly after IdU removal without HU treatment. Nascent strands were readily detected and the density of labeled fibers was similar in mock and HU-treated cells. The length of labeled tracts was measured in over 350 randomly chosen DNA fibers per sample condition. The median length of labeled DNA tracks did not decrease following HU addition (Fig 3B). We conclude that nascent DNA strands are not selectively targeted for degradation in HU-treated cells. While this experiment does not rule out the possibility that DNA is degraded non-selectively, the rapid resumption of DNA synthesis following HU removal (S4 Fig), indicated that the overall integrity of chromosomes was maintained.

**Fig 4:**
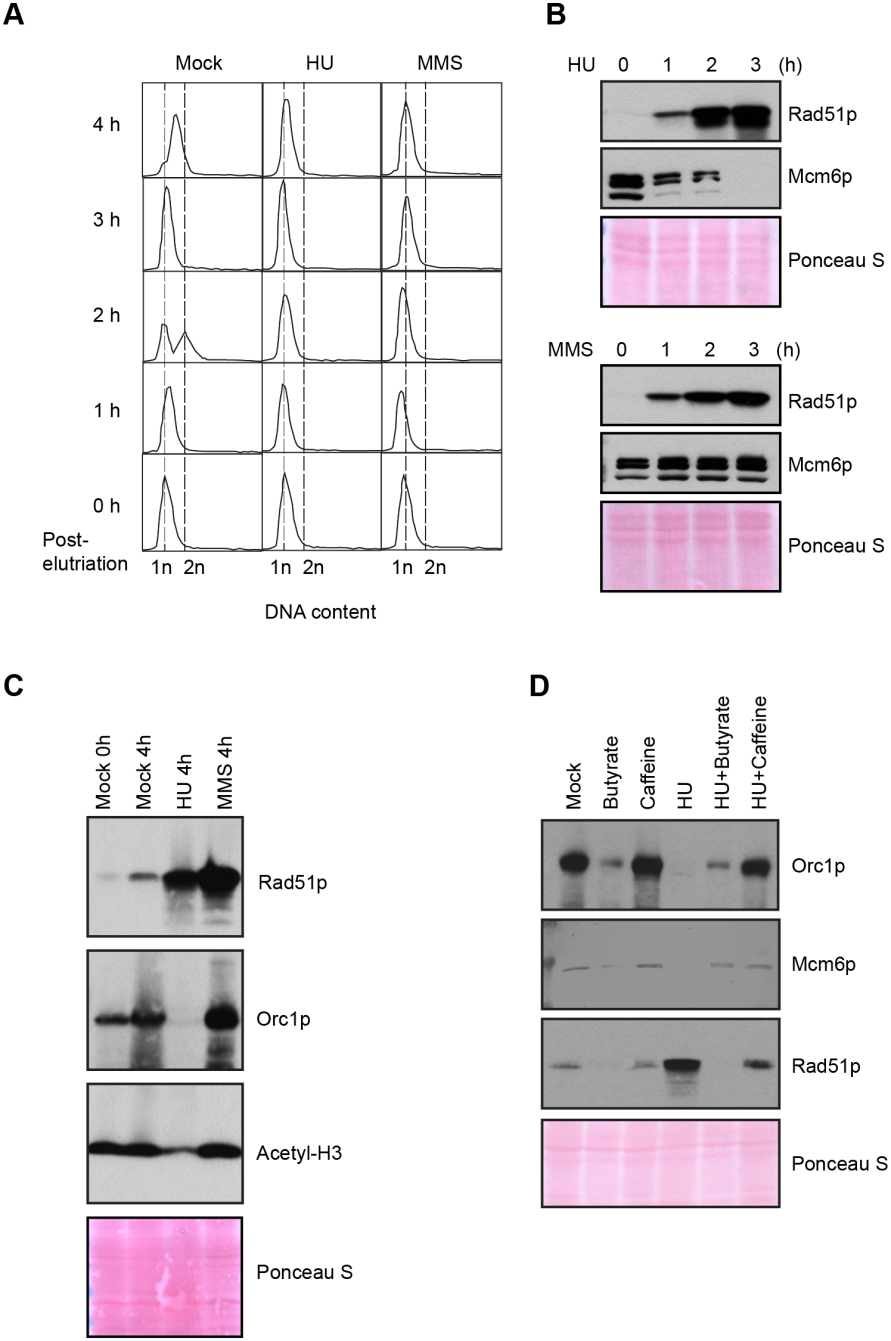
Effect of HU and MMS on pre-RC proteins in G1 treated cultures. HU (20 mM) or MMS (0.06%) were added immediately to an elutriated G1 phase population and cells were cultured for 4 h. (A) Flow cytometry analysis at 1 h intervals. (B) Western blot analysis with Rad51p and Mcm6p antibodies. Twenty micrograms of protein was loaded in each lane, as determined by Lowry protein assay, and Ponceau S staining was used to assess protein transfer prior to antibody probing. (C) Western blot analysis with Rad51p, Orc1p and acetylated histone H3 antibodies. Cells were synchronized by starvation and re-feeding, and cultured for 4 h +/- HU. (D) Western blot analysis of cells treated with 20 mM HU in the presence or absence of ATR and histone deacetylase inhibitors (1 mM caffeine and 50 mM sodium butyrate, respectively). For panel D, cells were synchronized by starvation and re-feeding, and subsequently cultured for 4 h. Western blot analysis with antibodies directed against Orc1p, Mcm6p and Rad51p.

Since DNA replication, DNA repair and chromatin compaction are influenced by epigenetic modifications, we next asked whether HU treatment affected two relevant post-translational histone modifications, phosphorylation of histone H2.AX and acetylation of histone H3. Phospho-gamma H2A.X accumulates at stalled replication forks in yeast and metazoa [1], and this modification is a reliable marker for activation of the *Tetrahymena* ATR checkpoint response [25]. Hyper-phosphorylation of H2A.X is associated with chromatin condensation, DNA fragmentation and apoptosis in metazoan cells [26]. Phospho-gamma H2A.X levels were elevated ~10-fold in HU-treated *Tetrahymena* (Fig 3C). Whereas, the ATR inhibitor, caffeine did not prevent H2A.X phosphorylation, the histone deacetylase (HDAC) inhibitor, sodium butyrate, lowered the gamma H2A.X induction ~2-fold (Fig 3C, gamma H2A.X). Moreover, sodium butyrate inhibited the accumulation of Rad51p in HU-treated cells, and was more effective than caffeine (Fig 4D). These results suggest that histone acetylation might have a protective function.

To explore potential links between HU and histone acetylation in *Tetrahymena*, we directly examined histone H3 with an acetylation-specific monoclonal antibody. Histone H3 acetylation levels were reproducibly reduced ~2-fold in HU-treated *Tetrahymena* (Figs 3C and 4C), and H3 hypo-acetylation was inhibited when sodium butyrate or caffeine were added at the time of HU addition (Fig 3C). We conclude that the chromatin landscape is altered in HU-treated *Tetrahymena*. We next asked whether hypo-acetylation contributes to the apparent decrease in DNA content in HU-treated cells. Consistent with this idea, the G1 peak failed to appear when sodium butyrate was added to mid-S phase HU-treated cells (Fig 3D). Instead, a very subtle, modest leftward shift in the mid-S phase DNA content peak was observed in HU + butyrate-treated cells.

The cumulative data suggest that chromatin compaction contributes significantly to the altered (G1-like) DNA profile in HU-treated *Tetrahymena*. Since H3 hyper-acetylation promotes precocious activation of late firing and dormant replication origins in other species [27–29], it is plausible that HU-induced hypo-acetylation could generate the opposite effect in *Tetrahymena*, inhibiting origin firing as part of a unique intra-S phase checkpoint response.

### ORC and MCM proteins are degraded during HU-induced replication stress

**Fig 5:**
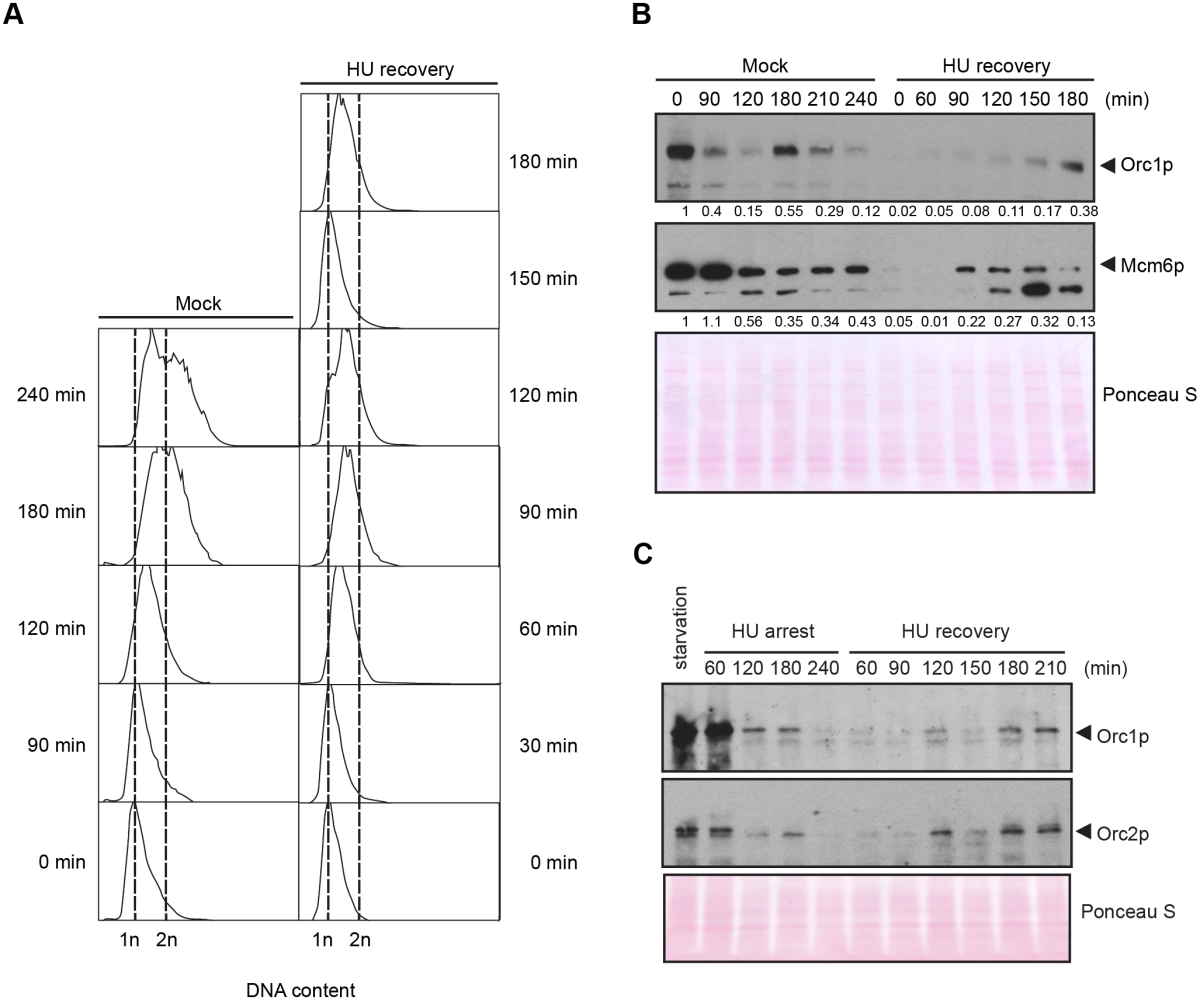
DNA replication and pre-RC replenishment following HU removal. A G1 population, synchronized by starvation and re-feeding, was treated with 20 mM HU for 4 h in 2% PPYS media. Cells were washed twice with HU-free media, re-suspended in their original volume and cultured for an additional 3 h in media lacking HU. Mock-treated, starved/re-fed cells were used as a control. (A) Flow cytometry analysis of starved/re-fed controls (left panel, mock) and HU-treated cells following drug removal (right panel, HU recovery). (B) Western blot analysis of the panel A time course with Orc1p and Mcm6p antibodies. Note the cell cycle oscillation of Orc1p in mock-treated cells, and the absence of Orc1p replenishment prior to completion of the first cell cycle in the HU recovery population. Twenty micrograms of protein was loaded in each lane, as determined by Lowry protein assay, and Ponceau S staining was used to assess protein transfer prior to antibody probing. (C) Western blot analysis of Orc1p and Orc2p during exposure to 20 mM HU in growth media (HU arrest), and following HU removal (HU recovery). Cells were synchronized by starvation and re-feeding. Twenty micrograms of protein was loaded in each lane.

In recently published work we showed that ORC and MCM protein levels change dramatically throughout development, and are co-regulated in vegetative growing cells [18]. To better understand the S phase checkpoint response, the fate of ORC and MCM proteins was examined in synchronized cell populations treated with HU or MMS. Western blot analysis revealed that the abundance of the Mcm6p remained relatively constant when MMS was added at the G1/S border or during mid-S phase (Fig 1B, G1 and S phase drug addition; Fig 4B, lower panel). This observation is consistent with yeast and higher eukaryotes, where MCM activity is regulated by reversible phosphorylation [7,30]. HU-treated cells behaved very differently. Mcm6p levels rapidly declined (Fig 1B, G1 and S phase HU addition; Fig 4B). More remarkably, Orc1p (Figs 4C and 5C, HU arrest) and Orc2p (Fig 5C, HU arrest) levels decreased in a time-dependent manner. Degradation of Orc1p was inhibited by the addition of caffeine to HU-treated cells (Fig 4D), implicating the involvement of ATR. Rad51p was modestly induced in HU + caffeine treated cells, consistent with a role for ATR (Fig 4D). We conclude that *Tetrahymena* replication stress (HU) and DNA damage (MMS) checkpoint responses differ in fundamental ways. Two questions immediately arose: are HU-treated cells competent to re-enter the cell cycle and faithfully replicate their chromosomes? What events must occur for ORC and MCM-depleted cells to progress through S phase?

### DNA replication precedes ORC and MCM replenishment during HU recovery

The precipitous drop in ORC and MCMs raised the possibility that origin licensing might be irreparably compromised in HU-treated cells. We first examined this possibility in cells that had transitioned past the G1/S border prior to cell cycle arrest. Synchronized S phase cells were exposed to HU for 4 h, and subsequently released into drug free media (recovery period). Flow cytometry was used to monitor cell cycle progression, and western blotting assessed the abundance of Rad51p and Mcm6p. Remarkably, the recovering cell population quickly resumed DNA replication and completed S phase within 2 h (S4A Fig), prior to the replenishment of Mcm6p (S4B Fig, 0–2 h, no caffeine). These cells replicated, divided and enter a second S without delay, as Mcm6p levels were eventually restored (S4B Fig, 4 h, no caffeine).

DNA synthesis and cell cycle progression during the recovery phase did not require inhibition of the ATR pathway, although the first cell cycle proceeded more quickly in caffeine-treated cells (S4A Fig). Whereas Rad51p levels were relatively constant during the recovery period, Rad51p levels declined and Mcm6p levels were replenished more quickly when caffeine was added (S4B Fig). Moreover, suppression of ATR was not required for new DNA synthesis, in spite of the high levels of damage sustained during HU exposure (Fig 3C, phospho-gamma H2A.X).

We next addressed the requirements for ORC during the first S phase following HU removal. G1-arrested cells were used as the starting point for two reasons. First, since Orc1p levels naturally cycle within an unperturbed cell population (Fig 5B, mock-treated control), peaking at G1 and disappearing by late S phase, we could effectively eliminate this variable by adding HU to G1 synchronized cells. Second, this regimen allowed us to follow an entire cell cycle after drug removal. Mock-treated starved/re-fed cells generated a characteristic DNA cell cycle profile (Fig 5A). They entered S phase and down regulated Orc1p within 90 min, and synthesized new Orc1p during G2 phase (180 min) (Fig 5B). The flow cytometry profile is indicative of a healthy synchronous cell cycle. By comparison, DNA content increased within 60 min after HU removal and a new G1 phase population appeared prior to the replenishment of ORC (Fig 5A and 5B, T = 120 min, T = 150 min). In a separate experiment, we probed for Orc2p. Orc1p and Orc2p were down regulated in HU-treated cells (Fig 5C). Like Orc1p, Orc2p levels did not increase prior to the completion of the first S phase during the recovery from HU-induced replication stress. The unexpected behavior of pre-RC components raised the possibility that DNA replication might occur by an ORC-independent mechanism.

### The rDNA replication origin is inactive and is passively replicated in ORC-depleted cells

**Fig 6:**
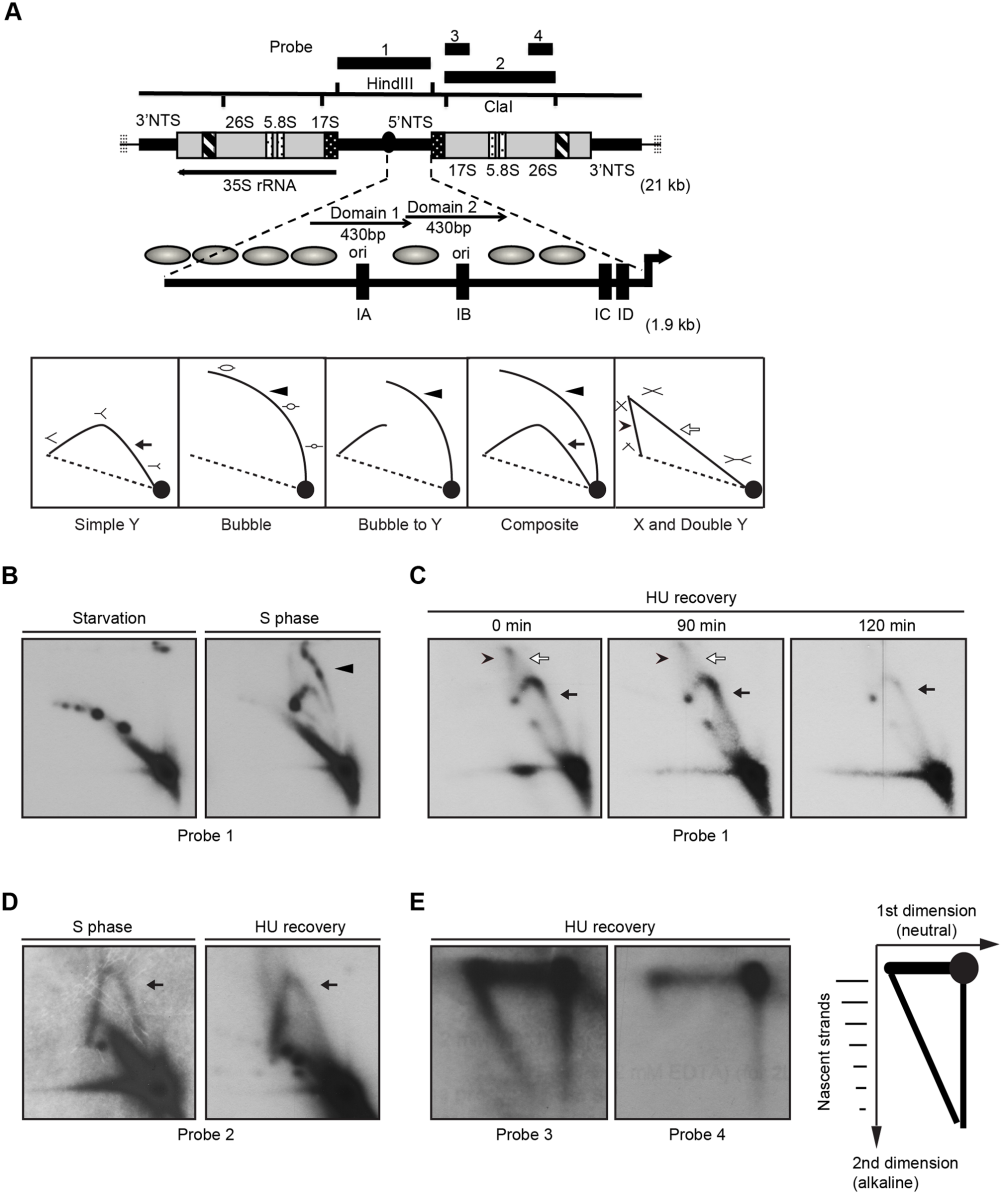
Two-dimensional gel electrophoresis analysis of rDNA replication intermediates following removal of HU. Cells were synchronized at the G1/S border by starvation and cultured in growth media in the absence or presence of 20 mM HU. DNA was prepared from mock-treated starved cells, mock-treated S phase cells, and HU-treated cells at defined intervals following HU removal. (A) Upper diagram: schematic of the 21 kb rDNA minichromosome and location of relevant restriction sites and probes for Southern blot analysis. Macronuclear rDNA minichromosomes contain two copies of the rRNA coding region and adjacent 5' and 3 'non-transcribed spacer (NTS) regions in an inverted orientation. The 35S rRNA precursor encodes the 17S, 5.8S and 26S rRNAs (grey areas- mature RNA coding regions, black and white stippled areas- processed RNA precursor regions, hatched area- self-splicing 26S rRNA intron). Telomeric DNA repeats (vertical hashes) are present at chromosome termini. The positions of four probes for N/N and N/A 2D gel analysis are shown. Expanded view of the 1.9 kb 5 NTS. Thick arrow- rRNA promoter; grey ovals- position of phase nucleosomes in vegetative rDNA minichromosomes [60], black boxes- type 1 repeats. Domains 1 and 2 (thin arrows) are 430 bp imperfect tandem repeats with 230 bp nuclease hypersensitive regions Lower diagram: schematic of typical RI patterns detected by N/N 2D gel electrophoresis. Simple Y arc (arrow): passive replication of the probed DNA fragment interval due to initiation elsewhere in the chromosome. Bubble arc (arrowhead): initiation at a central position in the probed DNA fragment. Bubble-to-Y arc: initiation at an asymmetric position in the examined fragment (low MW RIs: bubble arc (arrowhead), high MW RIs: Y arc). Composite: active (complete bubble arc, arrowhead) and passive (complete Y arc, arrow) replication within the probed DNA fragment. X and Double Y: the X spike (arrowhead) is generated from branch migration recombinant intermediates, and the double Y (open arrow) is generated by two converging replication forks. (B) RI patterns detected with the 5’ NTS probe 1 on *HindIII* digested DNA from mock treated quiescent (starvation) and replicating cell populations (S phase). (C) 5’ NTS analysis on DNA from HU-treated cells 0–120 min after HU removal (arrow: simple Y arc, passive replication; arrowhead: X-spike recombination intermediates). See flow cytometry profiles (Fig 5A) and western blots (Fig 5B) for cell cycle progression and abundance of Orc1p and Mcm6p, respectively. (D) Two-dimensional N-N gel analysis of the 5.5 kb rRNA coding region *ClaI* fragment (position 2168–7629, probe 2). (E) Two dimensional neutral-alkaline (N/A) analysis of RIs derived from the rRNA coding region *ClaI* fragment. Probe 3 spans nucleotides 2169 to 3670, and probe 4 spans nucleotides 5214–6676. Schematic of nascent-strand RIs resolved by N/A 2D gel electrophoresis. The 1n spot corresponds to non-replicating DNA. The vertical smear is derived from nicked, non-replicating DNA, and the horizontal smear represents the parental strand in RIs of different length. The diagonal arc corresponds to nascent-strand replication intermediates that are liberated from the parental strand by alkali denaturation prior to electrophoresis in the second dimension.

The *Tetrahymena* rDNA minichromosome serves as an excellent substrate to examine the effect of depleting ORC subunits. As shown previously, wild type strains initiate replication exclusively from nucleosome-free regions in the 5’ non-transcribed spacer (NTS) in this palindromic 21 kb minichromosome (Fig 6A, Domain 1 and Domain 2) [31]. ORC recruitment to rDNA origins is mediated by an integral RNA subunit, designated 26T RNA, that undergoes Watson-Crick base pairing with DNA sequences (type I elements) that are present at rDNA origins, but are absent from non-rDNA chromosomes [32,33]. Mutations in 26T RNA inhibit ORC recruitment to Domain 1 and Domain 2 origins [32]. Under these conditions, this origin is silenced, but the rDNA minichromosome is still replicated. Hence, our prior studies indicated that a backup program can be called into play when the normal mechanism for ORC-recruitment is compromised.

Neutral/neutral (N/N) two-dimensional gel electrophoresis was used to assess rDNA origin activity in ORC-depleted *Tetrahymena* during the recovery from HU-induced replication stress. A characteristic bubble-to-Y arc RI pattern was observed in the *Hind*III 5’ NTS fragment of mock-treated S phase cells (Fig 6B, S phase, probe 1, arrowhead identifies the bubble arc). This pattern was not detected in ORC-depleted cells after release from the HU block. Instead, the predominant RI pattern that was generated corresponds to a simple Y arc (Fig 6C, 0–120 min; filled arrow), indicative of passive replication of the 5’ NTS origins. A faint double Y arc pattern (open arrow) was also detected during the HU recovery phase, consistent with converging replication forks emanating from initiation events near the rRNA promoter or downstream of the 5’ NTS. Finally, an X spike was detected (arrowhead), indicative of branch migration in stalled replication forks or DNA molecules generated by homologous recombination. We conclude that the rDNA origin is inactivated in ORC-depleted cells.

To test for cryptic origins in the transcribed region, the 5.5 kb *ClaI* coding region fragment was examined (Fig 6A, schematic). No bubble arcs were detected in this region in mock-treated controls or cells recovering from HU-induced replication stress. Instead, a simple Y arc was observed (Fig 6D, probe 2). To examine this question further, neutral/alkaline (N/A) gel electrophoresis was used to visualize nascent strands and determine the direction of replication fork progression through this coding region interval. N/A analysis can distinguish between four possibilities: random initiation, initiation from cryptic origins in the coding region, initiation from more distal sites (3’ NTS or the telomere), and initiation from the promoter-proximal region. Whereas both short and long nascent strands were detected with 5’ NTS proximal probe 3, only high molecular weight RIs were detected with the more 3’ proximal probe 4 (Fig 6E). The collective data suggest that replication initiates near the rRNA promoter during the HU recovery phase. Further localization of the initiation site could not be achieved due to physical limitations of these methods and the absence of informative restriction sites to bracket the rRNA promoter.

### New initiation events predominate in ORC-depleted non-rDNA chromosomes

**Fig 7:**
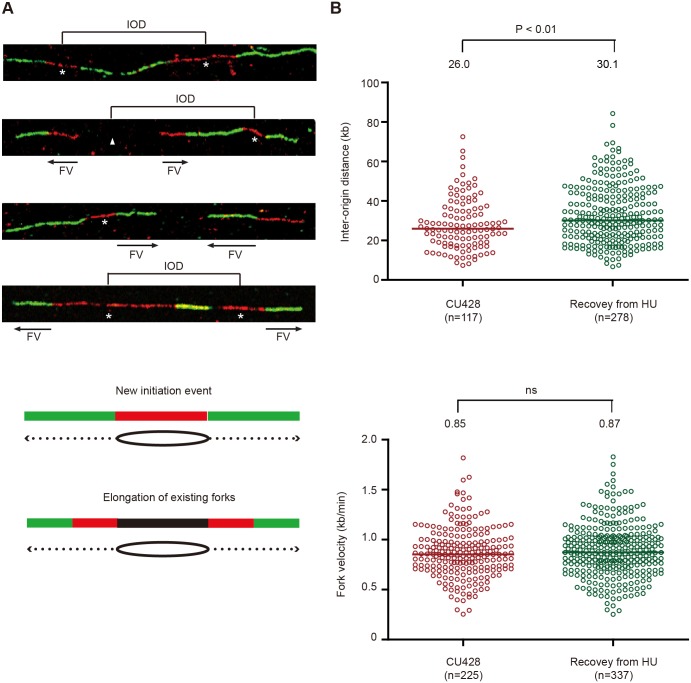
DNA fiber analysis of mock-treated cells and cells recovering from HU-induced replication stress. An asynchronous cell population was used to measure inter-origin distances and fork rates in mock-treated cells by sequential labeling with IdU and CldU for 10 min each (see Materials and Methods). HU-treated cells were synchronized at the G1/S border by starvation and re-feeding, and released into 20 mM HU for 4 h. Cells were washed free of HU, and incubated for 60 min prior to the sequential addition of IdU and CldU for 10 min each. (A) Representative DNA fibers images. (B) Compilation of DNA fiber image data and statistical analysis. Inter-origin distance was defined as the distance between the centers of two red segments in either green-red-green-red-green or green-red-gap-red-green tracks. Fork velocity was determined by measuring the length of the green segment in red-green tracks or the red segments in green-red-gap-red-green tracks. GraphPad Prism software was used to analyze the statistical significance, and the p-values shown in figures were determined by two-tailed unpaired t-test.

DNA fiber analysis was used to expand our analysis of DNA replication to the rest of the macronuclear genome during the HU-recovery phase. Most importantly, this method allowed us to distinguish between two models for genome-wide replication during the recovery phase: elongation of stalled forks generated prior to HU removal versus new initiation events. Cells were synchronized in G1 by starvation and re-feeding, and HU was immediately added to the media. HU was removed 4 h later and nascent DNA strands were sequentially labeled with IdU and CldU. New initiation events will generate DNA fibers in which the red signal (IdU pulse) is flanked by green (CldU, chase) on both sides. Elongation of existing forks will produce a green-red-gap-red-green pattern, in which the gapped region was replicated prior to the pulse/chase (HU recovery) period. Both patterns were detected following HU removal, prior to replenishment of ORC and MCMs (Fig 7A). New initiation events predominated, comprising ~80% of randomly chosen images. We conclude that bidirectional replication initiation is the predominant mechanism for genome-wide duplication of macronuclear chromosomes during the recovery from HU-induced replication stress.

DNA fibers were also used to quantitatively examine replication initiation and fork elongation in mock-treated controls (high ORC and MCMs) and during the first S phase after HU removal (low ORC and MCMs). The physical distance between replication initiation sites (inter-origin distance, IOD) and the speed of elongating replication forks (fork velocity, FV) were assessed [34]. In previously published work, we reported a median inter-origin distance of 24.3 kb and fork elongation rate of 0.83 kb/min for mid-log phase vegetative cells. An experimentally-induced 5-fold reduction in Orc1p and corresponding drop in Mcm6p had no effect on inter-origin distance, but resulted in a decreased fork elongation rate [18]. We performed a similar analysis on cells recovering from HU-arrest, prior to the replenishment of Orc1p, Orc2p and Mcm6p. Whereas the fork elongation rates for mock and HU-recovering cells were indistinguishable (Fig 7B), the median inter-origin distance increased by 16% during the first S phase following HU removal (from 26.0 kb to 30.1 kb; p<0.01). Hence, although the conventional replication initiation program is perturbed (Fig 6), new initiation events are still the major contributor to genome-wide replication under low ORC conditions.

## Discussion

The failure to completely replicate chromosomes leads to genome instability. While aberrant DNA replication decreases fitness, aneuploidy can facilitate unchecked cell proliferation by altering the expression of proto-oncogenes and tumor suppressor genes [35]. Previous studies in yeast and metazoa established that the ATR-dependent intra-S phase checkpoint response protects chromosomes by transiently arresting DNA replication through the reversible phosphorylation of the MCM2-7 complex [1]. Here, we describe a very different checkpoint response in the early branching eukaryote, *Tetrahymena thermophila*, in which essential pre-RC proteins (Orc1p, Orc2p, Mcm6p) are degraded. Upon removal of the cell cycle block, a full round of DNA replication occurs prior to replenishment of ORC and MCM proteins. These findings not only expand the repertoire of eukaryotic checkpoint responses, they raise the possibility that DNA replication can initiate on a genome-wide scale through an alternative (non-ORC based) mechanism.

### Unique characteristics of the *Tetrahymena* cell cycle and checkpoint response

A distinguishing feature of ciliated protozoa is the presence of two functionally distinct nuclei within a single cytoplasm. During vegetative growth, the diploid, germ line micronucleus and polyploid, somatic macronucleus replicate at different stages of the cell cycle. Their respective chromosomes segregate at different times and by unrelated mechanisms. The mitotic micronucleus divides during macronuclear G1 and amitotic macronuclear division is coupled to cytokinesis. Since the macronucleus confers the cellular phenotype, macronuclear DNA damage or replication stress would be expected to decrease fitness. However, aberrant macronuclear division with lagging chromosomes, indicative of incomplete DNA replication, is readily tolerated [22,33]. The high copy number of macronuclear chromosomes (45 C) may serve as a buffer against errors in DNA replication and chromosome transmission. Indeed, the macronucleus self corrects for genic balances by re-replicating chromosomes [36,37] or jettisoning excess DNA in the form of chromatin extrusion bodies [24]. Within the context of these seemingly chaotic cell cycles, *Tetrahymena* employs an ATR checkpoint kinase to arrest DNA replication in response to damage or replication stress [16]. ATR is also required for micronuclear genome stability and plays a critical role in meiotic chromosome transmission [17].

In this study, we exploited our ability to isolate a pure unperturbed population of G1 phase cells to explore the consequences of inducing DNA damage or replication stress prior to and during S phase. As expected, the administration of MMS or HU midway through macronuclear S phase led to rapid cell cycle arrest (Fig 1C), induction of the break-induced repair protein, Rad51p (Fig 1A and 1B), and phosphorylation of gamma H2A.X (Fig 3C). However, the parallels with yeast and metazoa diverge here. The first unanticipated feature of the *Tetrahymena* checkpoint response emerged from flow cytometry analyses of cells treated with HU at mid-S phase. In contrast to other eukaryotes where DNA content remains constant or gradually increases during prolonged HU exposure [8,11], the apparent DNA content decreased in HU-arrested *Tetrahymena*, assuming a value indistinguishable from G1 phase cells (Figs 1C–1E and S1B). DNA fiber analysis indicated that macronuclear DNA was not actively degraded (Fig 3B) and microscopic analysis ruled out DNA elimination through CEBs (Fig 2H). Formation of the ‘G1 peak’ was blocked by the HDAC inhibitor, sodium butyrate (Fig 3D), suggesting that chromatin was reorganized in HU-arrested cells. Consistent with this idea, histone H3 acetylation was reduced in HU-treated *Tetrahymena* (Figs 3C and 4C), and the macronucleus was more compact than mock-treated G1 phase cells (Fig 2G). The collective data suggest that this apparent decrease in DNA content is a reflection of changes in the epigenetic organization of the macronucleus. By analogy, studies in diploid maize cells have demonstrated that chromatin compaction can diminish propidium iodide fluorescence intensity [38]. Weakened interactions between chromosomes and the nuclear envelope promote chromatin compaction in cultured human cells, manifesting as aggregated chromatin clusters [39]. While we cannot distinguish between cause and effect at this time, our data suggest that the altered properties of macronuclear chromosomes described above are closely associated with epigenetic modifications on a genome-wide scale.

The second distinguishing feature of the *Tetrahymena* checkpoint response emerged from western blot analysis of HU and MMS-treated cells. A cornerstone of the intra-S phase checkpoint response in yeast and higher eukaryotes is the reversible phosphorylation-dependent inhibition of the replicative helicase, through ATR/CHK1-dependent phosphorylation of Mcm2p and Mcm3p [2,40,41]. In stark contrast, a core component of the replicative helicase, Mcm6p, is degraded in HU-arrested *Tetrahymena* (Fig 4B). Moreover, Orc1p and Orc2p proteins are degraded. The decline in these proteins is not reflective of the natural progression through S phase. First, these proteins are degraded when HU is added prior to S phase entry. Flow cytometry and DNA fiber analysis corroborate the arrest of replication initiation and elongation in HU-treated *Tetrahymena* (Figs 3–5). Second, while Orc2p levels do not oscillate across the cell cycle [33], this protein is degraded in HU-treated cells (Fig 5C). Finally, although MMS and HU elicited intra-S phase checkpoint responses, MMS does not trigger ORC and MCM degradation (Fig 4B and 4C), irrespective of the time of addition.

The third and most unexpected behavior of the *Tetrahymena* checkpoint response was revealed through our analysis of DNA replication after HU removal: an entire S phase occurred without replenishment of ORC and MCM proteins (Figs 5 and S4). The sole origin in the rDNA minichromosome is silenced (Figs 6 and 7) and no cryptic origins were uncovered in the 21 kb rDNA minichromosome. However, leading strand synthesis proceeds toward the telomeres, suggesting that replication initiates at a new site proximal to the rRNA promoter. New initiation events are commonplace in non-rDNA chromosomes (Fig 7) and the inter-origin distance increases slightly, indicating that stalled forks play a minor role in the replication of chromosomes during the recovery phase.

Replication stress has been shown to trigger different, species-specific checkpoint responses and recovery mechanisms. For example, while HU inhibits replication initiation in *S*. *cerevisiae*, it only slows replication forks in *S*. *pombe* [8]. Furthermore, most late firing origins in *S*. *pombe* are regulated by a RAD3 (ATR) independent mechanism [11]. Although mammalian ATR inhibits replication initiation and elongation [9,10], replication origins are far from equivalent, since neighboring origins are differentially sensitive to the depletion of dNTP pools [42]. The unifying features in these species are the stabilization of late firing origins and reversible phosphorylation of MCM subunits [2–4]. Once the source of stress is removed, the conventional DNA replication program resumes [5,43].

We propose that the DNA replication properties of HU-treated *Tetrahymena* are a manifestation of DNA replication programs that normally operate during macronuclear development. Recent studies revealed that the abundance of ORC and MCM proteins are dynamically co-regulated [18]. Partial depletion of Orc1p by gene disruption leads to a concomitant decrease in Orc2p and Mcm6p levels during the vegetative stage of the life cycle. Moreover, ORC and MCM naturally fluctuate to a greater degree during conjugation, and poorly correlate with the demands for DNA replication: ORC and MCM are elevated early in development when the replication load is low, and precipitously decline when the replication load increases [18]. Aberrantly migrating (RNase A + mung bean nuclease-sensitive) RIs are generated in the endoreplicating macronucleus, suggesting that RNA-DNA hybrids accumulate, similar to yeast senataxin mutants [18,44]. Hence, the initiation of DNA replication is dynamically re-programmed during *Tetrahymena* development. Our analysis of cells recovering from HU-induced replication stress suggests that the capacity to replicate under low ORC and MCM conditions is retained during the vegetative phase of the life cycle.

### Models for replication initiation under limiting ORC conditions

Several models are considered for DNA replication with limiting amounts of ORC. In the first model, replication initiation and elongation are uncoupled in HU-treated *Tetrahymena*. Stalled replication forks generated during HU arrest are simply elongated during the recovery phase. Whereas initiation and elongation are transiently uncoupled in Drosophila follicle cells [45], two pieces of data disfavor this model in *Tetrahymena*. First, during the recovery from HU-induced stress, new initiation events were detected by 2D gel analysis in rDNA minichromosome; however, the 5’ NTS origin did not serve as the initiation site (Fig 6). More importantly, the predominant CldU/IdU/CldU pattern in non-rDNA DNA fibers generated during HU recovery corresponds to new initiation events rather than the elongation of stalled replication forks (Fig 7A).

In the second model, a subpopulation of origins is refractory to ORC and MCM depletion. Initiation events during HU recovery could occur at cryptic or late-firing origins, analogous to origins that are used by *S*. *cerevisiae* upon HU removal [46] This model predicts that these origins would function at low ORC concentrations, and consequently should have a high affinity for ORC [47]. *Tetrahymena*’s intrinsic ability to partially re-replicate the macronuclear genome, despite S phase-specific degradation of Orc1p, is consistent with this model [33,36]. A confounding factor is the global decrease in histone acetylation in HU-treated *Tetrahymena*. Based on studies in yeast [27], we would predict that this epigenetic state would disfavor ORC-dependent initiation events. Further studies are needed to determine the relative contribution of chromatin-associated and pre-deposition histones to the H3 acetylation profile in HU-arrested *Tetrahymena* [48].

A third model posits that the genome is duplicated in an ORC-independent manner. Archaebacteria and animal viruses provide the only precedents. *Haloferax volcanii* strains lacking all four ORC-dependent chromosomal origins exhibit increased fitness relative to strains with 1–4 intact origins [49]. Cell viability in the originless mutant is dependent on RadA recombinase, which is non-essential in wild type strains. In this view, the converging replication forks and branch migration intermediates in the *Tetrahymena* rDNA 5’ NTS (Fig 5B) could represent recombination substrates or intermediates that prime DNA replication. Based on the measured inter-origin distance (Fig 7), approximately 250,000 recombination events would be required to replicate a single polyploid macronucleus. Alternatively, DNA replication could be primed by a covalently bound protein, as in adenovirus [50], tRNA molecules (avian sarcoma virus) [51], or non-coding RNA. While there is no precedent for these scenarios on genome-wide scale, RNA polymerase I generated RNA-DNA hybrids (R loops) have recently been shown to prime unscheduled replication initiation in the rDNA of *S cerervisiae* [52]. Stable RNA-DNA hybrids have been detected in endoreplicating rDNA molecules, when *Tetrahymena* ORC concentrations are low [18]. Another possibility is that small non-coding RNAs could be involved. Dicer-generated 27–30 nt RNAs, direct the removal of ~6000 IES elements in the developing *Tetrahymena* macronucleus [53,54]. Small (23–24 nt) non-coding RNAs of unknown function are produced during the vegetative cell cycle [55]. The 287 nt long non-coding RNA, 26T RNA, is an integral component of ORC, selectively directing the complex to complementary DNA sequences at the amplified rDNA origin [32]. The underlying mechanism for replication initiation in ORC-depleted *Tetrahymena* awaits further studies.

The *Tetrahymena* system has several interesting unanticipated parallels in which endoreplication is a common theme. DSB-inducing agents can trigger an ATR-dependent endoreplication program in *Arabidopsis thaliana* rather than simply arrest the cell cycle [12]. While Drosophila ORC is normally present in endoreplicating nurse cells [56], ORC2-deficient mutant cells undergo multiple rounds of endoreplication by an undetermined mechanism [57]. The archaebacteria *H*. *volvanii*, which efficiently propagates originless chromosomes is polyploid, and can tolerate significant variation in chromosome copy number [49]. We propose that polyploid cells are inherently more tolerant than diploid cells to DNA damage and replication stress, and can duplicate their chromosomes by unconventional mechanisms that might also function to a lesser degree in haploid/diploid tissues and/or species. High-throughput mapping of replication origins should provide novel insights into underlying mechanism(s) for replication initiation site selection in *Tetrahymena*.

## Materials and Methods

### Cell cycle synchronization and DNA damage checkpoint activation

*Tetrahymena thermophila* strain CU428 was obtained from the *Tetrahymena* Stock Center. Standard methods were used to cultivate cells and visualize cells by light and fluorescence microscopy [16]. Cell cycle synchronization was achieved by starvation and re-feeding or by centrifugal elutriation as previously described [33]. For DNA damage checkpoint activation, standard growth media was supplemented with either 20 mM hydroxyurea (HU, Sigma Chemical) or 0.06% methylmethansulphonate (MMS, Sigma Chemical). Stock solutions of caffeine and sodium butyrate (Sigma-Aldrich) were prepared in water. The working concentrations of caffeine and sodium butyrate were 1 mM and 50 mM, respectively. For cell division analysis, DAPI-stained cells were examined microscopically at defined intervals after drug treatment and removal.

### Flow cytometry

Flow cytometry analysis was performed on Becton Dickinson FACSAria II flow cytometer (BD Biosciences), using propidium iodide to monitor DNA content. Data were analyzed using BD FACSDiva software. Histograms were plotted, in which the y-axis represents the number of events and the x-axis represents the relative DNA content. Forward and side scatter data were graphed to visualize the distribution of individual cells within the population.

### Western blot analysis

Whole cell extracts were prepared by lysing cells in 1% SDS lysis buffer (50 mM Tris at pH 8.0, 150 mM NaCl, 1 mM EDTA, 1% NP-40, 1% sodium deoxycholate, 1% SDS) for 15 min on ice. Protein concentrations were determined using the modified Lowry protein assay reagents (Bio-Rad). Unless otherwise stated, equal amounts of total protein (20 μg) were loaded in each lane and samples were subjected to SDS-PAGE and transferred to nitrocellulose membranes (Whatman Protran BA85, GE Healthcare). Prior to probing, membranes were stained with Ponceau S staining (Sigma-Aldrich) to confirm equivalent sample loading and protein transfer. Immunodetection of *Tetrahymena* Orc1p (1:5,000), Orc2 (1:5,000) and Mcm6p (1:10,000) were carried out using polyclonal rabbit antibodies raised against immunogenic *Tetrahymena* peptides (Covance). Antibodies directed against Rad51 (51RAD01, Thermo Scientific, 1:5,000 dilution), phospho-gamma H2A.X (2F3, BioLegend, 1:1,000 dilution), and acetyl-histone H3 (polyclonal antibody #06599, Millipore, 1:10,000 dilution) were obtained from the indicated commercial sources). Blots were incubated with the primary antibodies at 4°C overnight, washed and incubated for 3 h at 4°C with horseradish peroxidase-conjugated goat anti-rabbit or goat anti-mouse IgG (Jackson ImmunoResearch). Membrane bound secondary antibodies were visualized using ECL reagents (PerkinElmer) according to the manufacturer’s instructions. Densitometry of blots was performed using ImageJ software (version 1.47v for Macintosh, National Institutes of Health).

### Two-dimensional (2D) gel analysis of DNA replication intermediates

Total genomic DNA was isolated from *Tetrahymena* cultures as previously described to preserve DNA replication intermediates (RI) [58]. For RI enrichment, 200 μg of genomic DNA were digested with *HindIII* (rDNA 5’ NTS analysis) or *ClaI* (rDNA coding region analysis) for 4 h and applied to the 200-μl packed volume benzoylated naphthoylated DEAE (BND)-cellulose (Sigma-Aldrich). Caffeine-eluted DNA samples were precipitated with isopropanol, using 20 μg glycogen as a carrier. Total DNA recovery was estimated to be ~5% of input.

Neutral-neutral (N/N) and neutral-alkaline (N/A) 2D gel electrophoresis were performed as previously described [31]. Approximately 3–10 μg of BND cellulose-enriched DNA was loaded for each 2D gel experiment. Nor N/N analysis, the first dimension gel (0.4% agarose) was run in 1X TAE buffer (40 mM Tris, 20 mM acetic acid, and 1 mM EDTA) at 1.5 V/cm for 20 h at RT. The second dimension gel (1% agarose) was run in 1X TBE buffer (90 mM Tris, 90 mM boric acid, 2 mM EDTA) containing 0.5 mg/ml ethidium bromide at 3 V/cm for 18 h at 4°C. For N/A analysis, the second dimension separation was performed at 4°C in recirculating buffer (40 mM NaOH, 2 mM EDTA) at 1.5 V/cm for 20 h.

DNA was transferred overnight to a charged nylon membrane (Hybond-XL, Amersham) in alkaline buffer by capillary blotting. Membranes were prehybridized at 37°C for 4 h in 1M NaCl, 1% SDS, 10% dextran sulfate, 5 mM Tris [pH 7.5], 100 μg/ml of denatured salmon sperm DNA and 25% formamide. rDNA 5’ NTS and coding region probes were labeled by random priming and added directly to the prehybridization solution. After 18 h, membranes were washed three times in 2X SSC/1% SDS solution for 15 min each at 42°C, and once in 0.4X SSC/0.1% SDS solution for 15 min at 42°C. Blot were exposed to X-ray film with an intensifying screen at -70°C or analyzed with a phosphorimager.

### DNA fiber analysis

*Tetrahymena* was cultured in 2% PPYS media to the density of 1.5x10e5 cells/ml. For pulse chase experiments, cells were sequentially pulse labeled with 400 μM IdU (Sigma) and 100 μM CldU (MP Biomedicals) at 30°C. The media was removed and cells were washed once with 1X PBS between each labeling step. For DNA fiber processing, cells were washed twice with PBS, the cell density was adjusted to 1x10e6 cells/ml. Preparation and immunostaining of DNA fibers was performed as described previously described [59] with the following modifications. Briefly, after fixation and HCl treatment, slides were washed three times with 1X PBS, and blocked with 5% BSA in PBS for 30 min. Mouse anti-BrdU (1:50, Becton Dickson) and rat anti-BrdU (Accurate Chemical, 1:100 dilution) antibodies in 5% BSA were then added onto slides. After 1 h incubation, the slides were washed three times again with 1X PBS, and incubated for 30 min with secondary antibodies: Alexa Fluor 568 goat anti-mouse IgG (Invitrogen/Molecular Probes, 1:100 dilution) and Alexa Fluor 488 goat anti-rat IgG (Invitrogen/Molecular Probes, 1:100 dilution). Finally, slides were washed three times with 1X PBS, dehydrated with an ethanol series, and mounted with SlowFade Gold antifade solution (Invitrogen). During immunostaining, all antibodies were diluted in 5% BSA in 1X PBS, all incubations were performed at 37°C, and all wash steps were done at RT. DNA fiber images were obtained on a Nikon A1R+ confocal microscope at 600X magnification. Measurements of track length were performed with Nikon NIS-Elements software. Green-red-green tracks represent origins that initiated DNA replication during the IdU pulse. Green-red-gap-red-green tracts represent origins that initiated DNA replication prior to the IdU pulse. Inter-origin distance was defined as the distance between the centers of two red segments in either green-red-green-red-green or green-red-gap-red-green tracks. Fork velocity was determined by measuring the length of the green segment in red-green tracks or the red segments in green-red-gap-red-green tracks. GraphPad Prism software was used to analyze the statistical significance, and the p-values shown in figures were determined by two-tailed unpaired t-test.

## Supporting Information

S1 Fig Flow cytometry profiles of cells synchronized by starvation and re-feeding. (A) Mid-log phase cultures were placed into starvation media (10 mM Tris, pH 7.4) for 12 h to synchronize cells at the G1/S border. 5% PPYS was added to resume cell cycle progression. 20 mM HU or 0.06% MMS was added at the time of re-feeding or 3 h later (B), when cells were in mid-S phase, and DNA content was analyzed by flow cytometry. The arrow in panel B points to the G1 peak generated over time in HU and MMS-treated cells. Note the broad G2 peak that formed 4 h after re-feeding in mock controls did not appear throughout the entire HU or MMS time course. In other representative experiments, MMS-treated cells did not generate a pronounced G1 shoulder. Instead, the entire DNA content profile shifted to the left (lower apparent DNA content).
(TIF)

S2 Fig Flow cytometry analysis at 10 min intervals. Cells were synchronized at the G1/S border by centrifugal elutriation, and 20 mM HU was added 1 h later, when cells were in mid-S phase. DNA samples were collect for flow cytometry analysis. Note the pronounced G2 peak in mock-treated cells that appears after the time of HU addition (30/60/90 min). Whereas a G1 peak gradually formed in the HU-treated cells, none of the 25 samples in this time course (30–270 min) generated a G2 DNA content after HU addition.
(TIF)

S3 Fig Forward and side scatter side scatter flow cytometry parameters. (A) Mock, (B) G1 HU-treated, and (C) mid-S phase HU-treated cells were subjected to flow cytometry analysis at hourly intervals. Flow cytometry analyses: left panel- DNA content (PI intensity), right panel- plot of SSC (Y axis) versus FSC (X axis). HU treated cells exhibited an increase in SSC, regardless of the time of drug addition. A subset of these data are depicted in Fig 2E.
(TIF)

S4 Fig Recovery from HU-induced cell cycle arrest. Elutriated cells were allowed to progress to mid-S phase and 20 mM HU was added for 8 h. Cells were washed twice and resuspended in HU-free media supplemented with (+) or lacking 1 mM caffeine. Samples were taken at 1 h intervals. (A) Flow cytometry analysis of HU-arrested and released cells. (B) Western blot analysis of Rad51p and Mcm6p. Lower panel: Ponceau S staining of PVDF membranes prior to antibody probing.
(TIF)
