# Supplementary figures and images for: Checkpoint Activation of an Unconventional DNA Replication Program in Tetrahymena
Source: PLoS Genet. 2015 Jul 28;11(7):e1005405. doi: 10.1371/journal.pgen.1005405 (PMC4517752; doi:10.1371/journal.pgen.1005405)

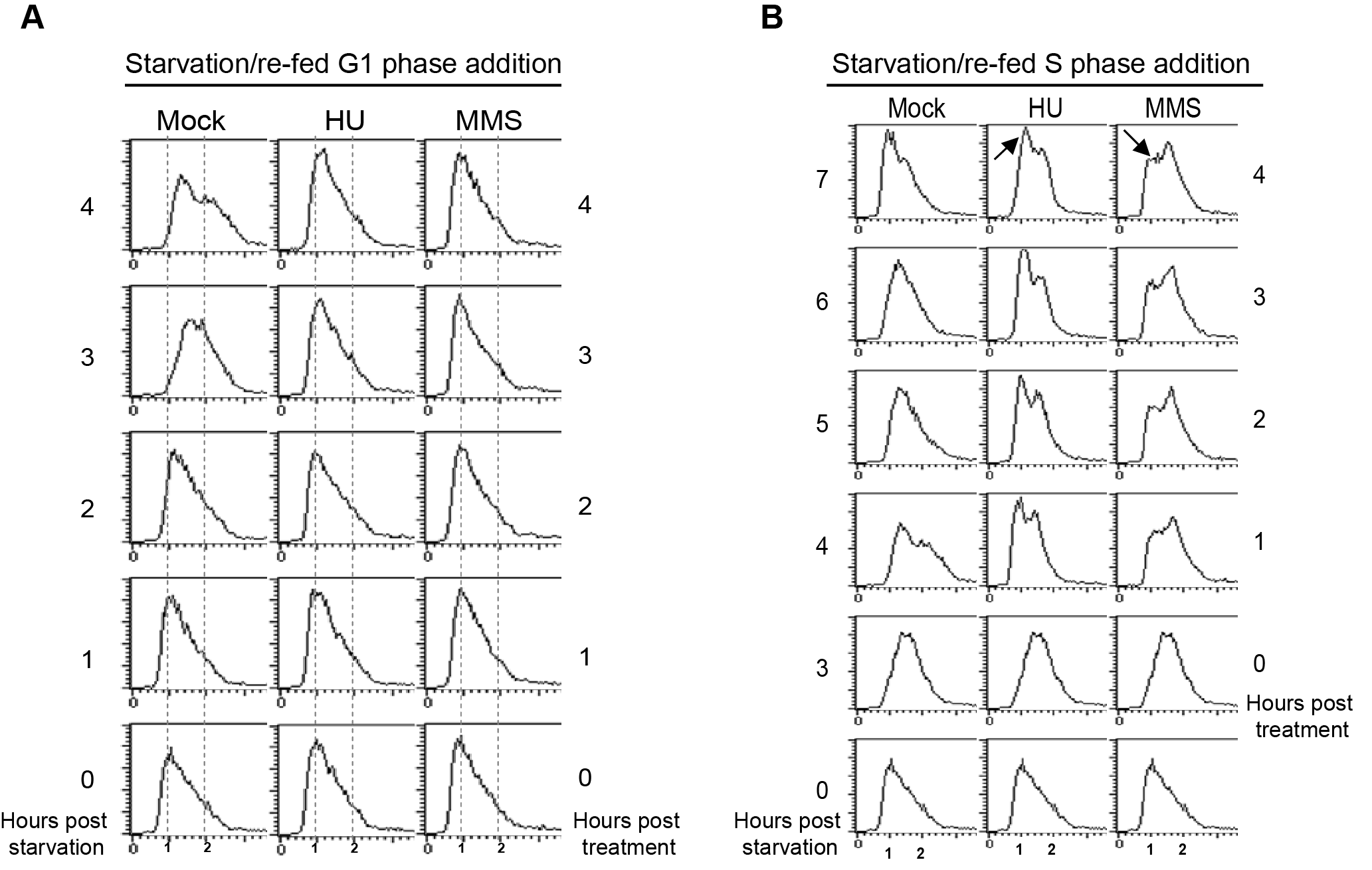

Supplement: S1 Fig — (A) Mid-log phase cultures were placed into starvation media (10 mM Tris, pH 7.4) for 12 h to synchronize cells at the G1/S border. 5% PPYS was added to resume cell cycle progression. 20 mM HU or 0.06% MMS was added at the time of re-feeding or 3 h later (B), when cells were in mid-S phase, and DNA content was analyzed by flow cytometry. The arrow in panel B points to the G1 peak generated over time in HU and MMS-treated cells. Note the broad G2 peak that formed 4 h after re-feeding in mock controls did not appear throughout the entire HU or MMS time course. In other representative experiments, MMS-treated cells did not generate a pronounced G1 shoulder. Instead, the entire DNA content profile shifted to the left (lower apparent DNA content). (TIF) [file pgen.1005405.s001.tif]

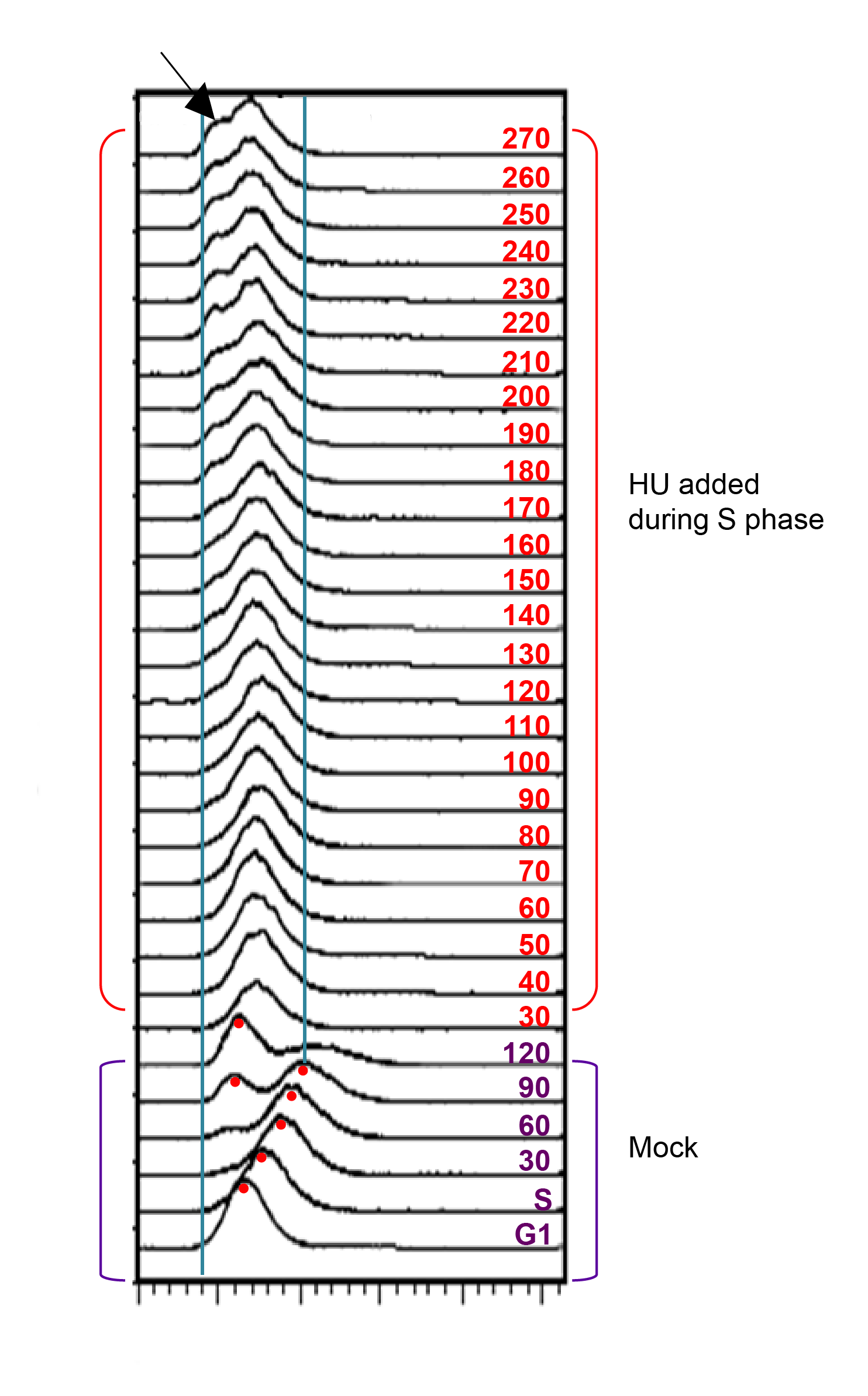

Supplement: S2 Fig — Cells were synchronized at the G1/S border by centrifugal elutriation, and 20 mM HU was added 1 h later, when cells were in mid-S phase. DNA samples were collect for flow cytometry analysis. Note the pronounced G2 peak in mock-treated cells that appears after the time of HU addition (30/60/90 min). Whereas a G1 peak gradually formed in the HU-treated cells, none of the 25 samples in this time course (30–270 min) generated a G2 DNA content after HU addition. (TIF) [file pgen.1005405.s002.tif]

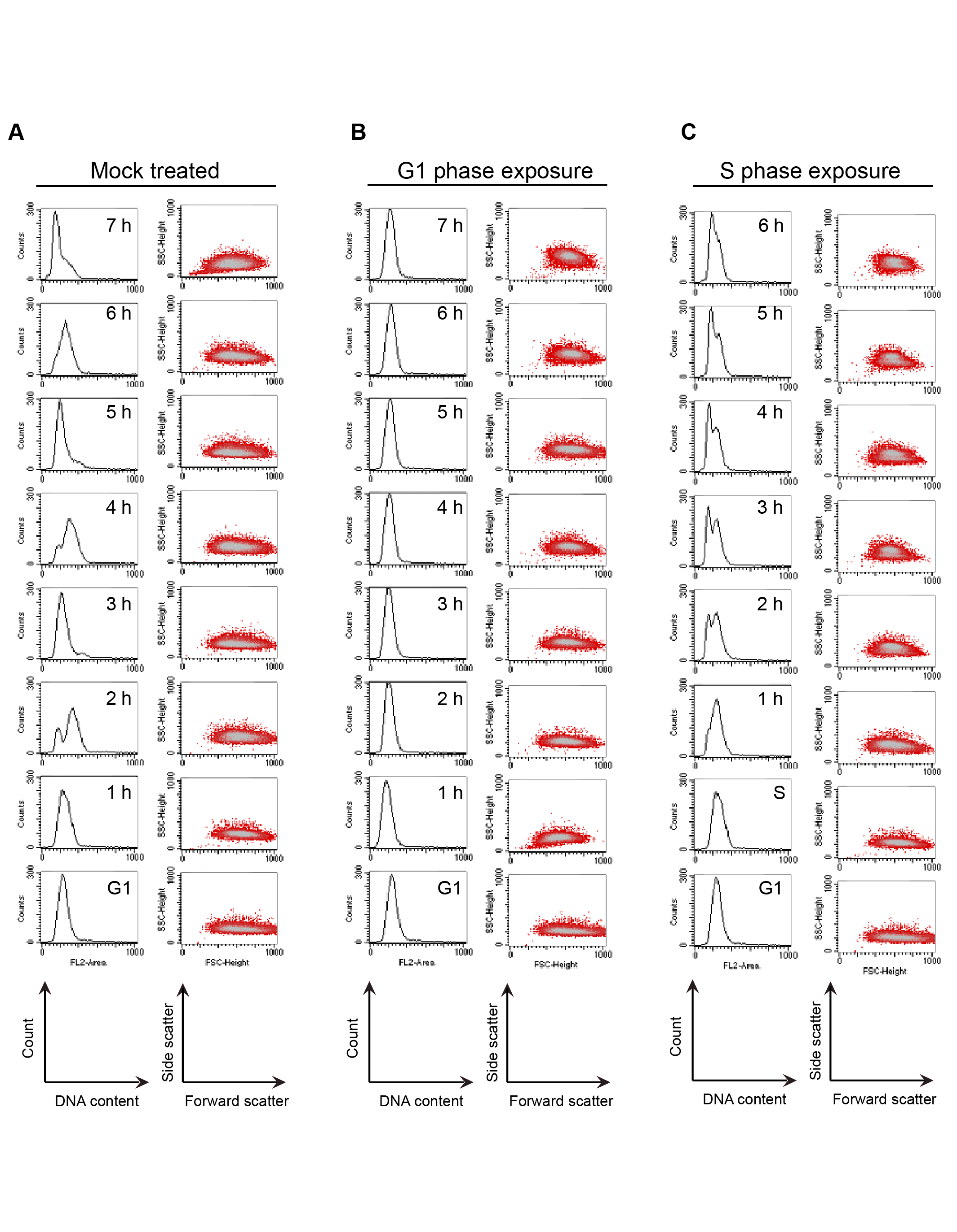

Supplement: S3 Fig — (A) Mock, (B) G1 HU-treated, and (C) mid-S phase HU-treated cells were subjected to flow cytometry analysis at hourly intervals. Flow cytometry analyses: left panel- DNA content (PI intensity), right panel- plot of SSC (Y axis) versus FSC (X axis). HU treated cells exhibited an increase in SSC, regardless of the time of drug addition. A subset of these data are depicted in Fig 2E. (TIF) [file pgen.1005405.s003.tif]

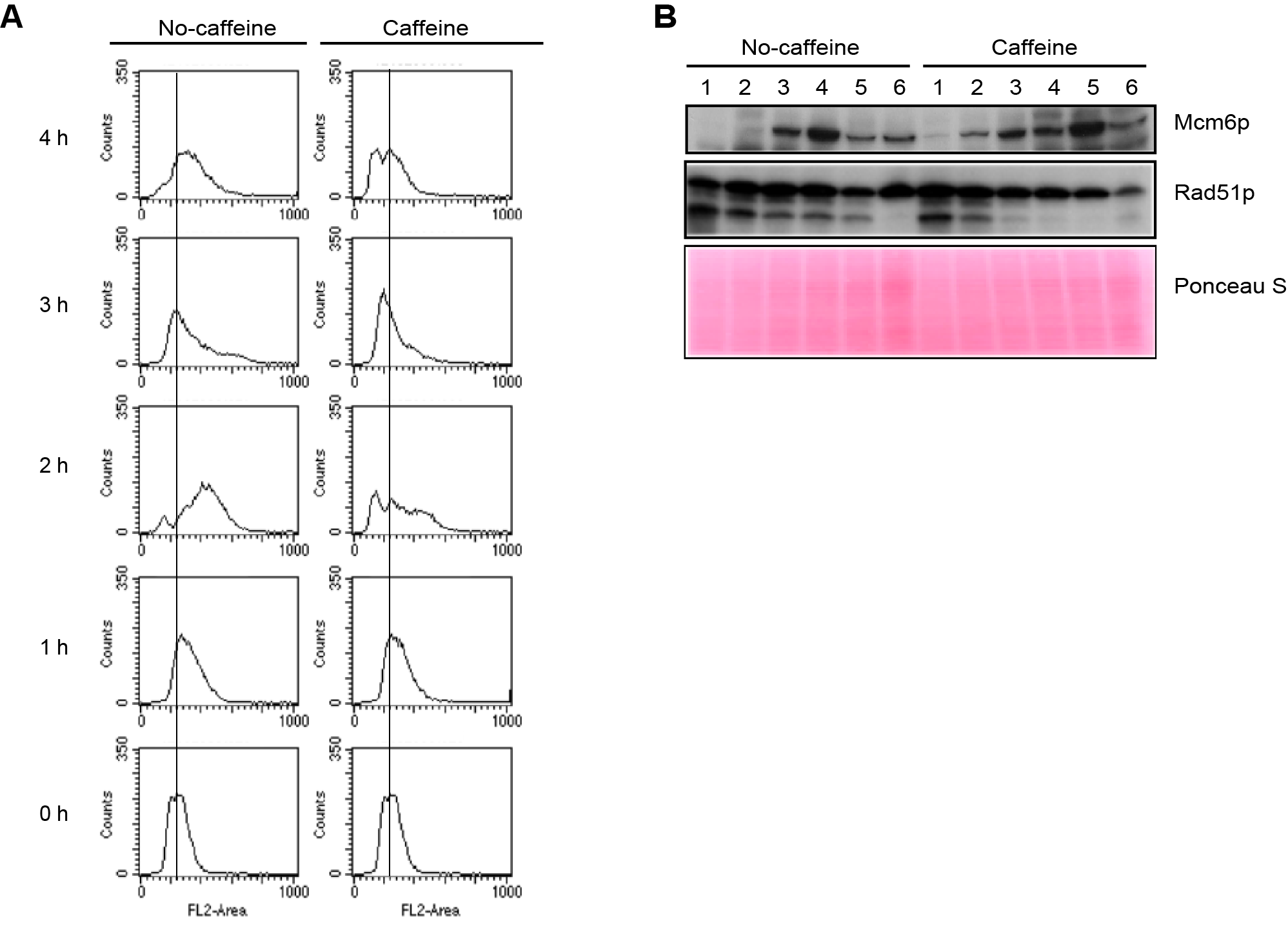

Supplement: S4 Fig — Elutriated cells were allowed to progress to mid-S phase and 20 mM HU was added for 8 h. Cells were washed twice and resuspended in HU-free media supplemented with (+) or lacking 1 mM caffeine. Samples were taken at 1 h intervals. (A) Flow cytometry analysis of HU-arrested and released cells. (B) Western blot analysis of Rad51p and Mcm6p. Lower panel: Ponceau S staining of PVDF membranes prior to antibody probing. (TIF) [file pgen.1005405.s004.tif]
